# *Drosophila Melanogaster*

**DOI:** 10.1002/1097-0061(20000630)17:2<146::AID-YEA24>3.0.CO;2-A

**Published:** 2000

**Authors:** Jo Wixon, Cahir O'Kane

**Affiliations:** 1 Managing Editor School of Biological Sciences University of Manchester Oxford Road Manchester M13 9PT UK; 2 Section Editor (Drosophila) Department of Genetics University of Cambridge Downing Street Cambridge CB2 3EH UK


**Yeast**
*Yeast* 2000; **17**: 146–153.

**Featured Organism**

### *Drosophila melanogaster*

Jo Wixon[1] and Cahir O'Kane[2]
[1] *Managing Editor, School of Biological Sciences, University of Manchester, Oxford Road, Manchester M13 9PT, UK*
[2] *Section Editor (Drosophila), Department of Genetics, University of Cambridge, Downing Street, Cambridge CB2 3EH, UK.*

### Summary

- **3 mm long dipteran (fly), commonly known as the fruit fly.**
- **Adult fly is yellow-brown in colour, with oval wings and rust-red eyes.**
- **~180 Mb genome, as four chromosomes X/Y, 2, 3 and 4.**
- **Genome sequence substantially completed 24 March 2000.**
- **120 Mb euchromatic region of genome comprises 13 601 predicted genes.**
- **Has made significant contribution to understanding of development.**
- **Large-scale P-insertion mutagenesis (and enhancer-trap), microarray and expression pattern projects underway, proteomics project being set up.**
- **Comparative studies with insect relatives including grasshoppers, beetles and moths, but mainly used in comparative studies with higher eukaryotes.**

### Background

*Drosophila* melanogaster is a fruit fly, which is about 3 mm long and has a short life cycle of just 2 weeks. The *Drosophila* egg is about 0.5 mm long. At 25°C, the embryo can develop and hatch into a worm-like larva about a day after fertilization. Larvae eat and grow continuously, the first instar moults 1 day after hatching, then the second instar moults again after another day, to form the third and final larval instar. The fly remains as a third instar larva for 2 days, then forms an immobile pupa. The body is completely remodelled to produce the adult, winged form during the next 4 days. The adult then hatches from the pupal case and is fertile after another day. The adult flies are a yellow-brown colour, with oval wings and rust-red eyes. The female is slightly larger than the male and the two sexes can be easily distinguished by the black markings on their abdomens.

As a fly larva grows, the number of cells that form differentiated larval tissues remains constant, but more gene products are required. To cope with this demand, each chromosome divides hundreds of times and the cells increase in size. Since all the replicated strands remain attached to each other, this results in massively thick polytene chromosomes, which can be seen clearly using a microscope. Polytene chromosomes have unique patterns of dark and light bands, which can be used to generate a map, onto which nucleic acid probes (of individual cloned genes, for example) can be placed. The standard map of the polytene chromosomes has 102 numbered bands. These are further divided into six lettered bands (A–F) which, on average, each contain about 300 kb of DNA and 15–25 genes.

*Drosophila* shares many conserved genes with humans. However, its usefulness as a model system goes beyond simple comparison of genes, but encompasses the study of many cellular and developmental processes. In addition to similarities in basic cellular structure and function, humans and *Drosophila* share pathways for intercellular signalling (Pawson and Bernstein, 1990), developmental patterning (Krumlauf, 1992), learning and behaviour (Kandel and Abel, 1995) and tumour formation and metastasis (Potter *et al*., 2000). The list of similarities is still expanding to include phenomena like neuronal degeneration (Fortini and Bonini, 2000), behavioural effects of drugs and neurotransmitters (Bainton *et al*., 2000; Li *et al*., 2000) and sleep (Shaw *et al*., 2000; Hendricks *et al*., 2000).

Copyright © 2000 John Wiley & Sons, Ltd.

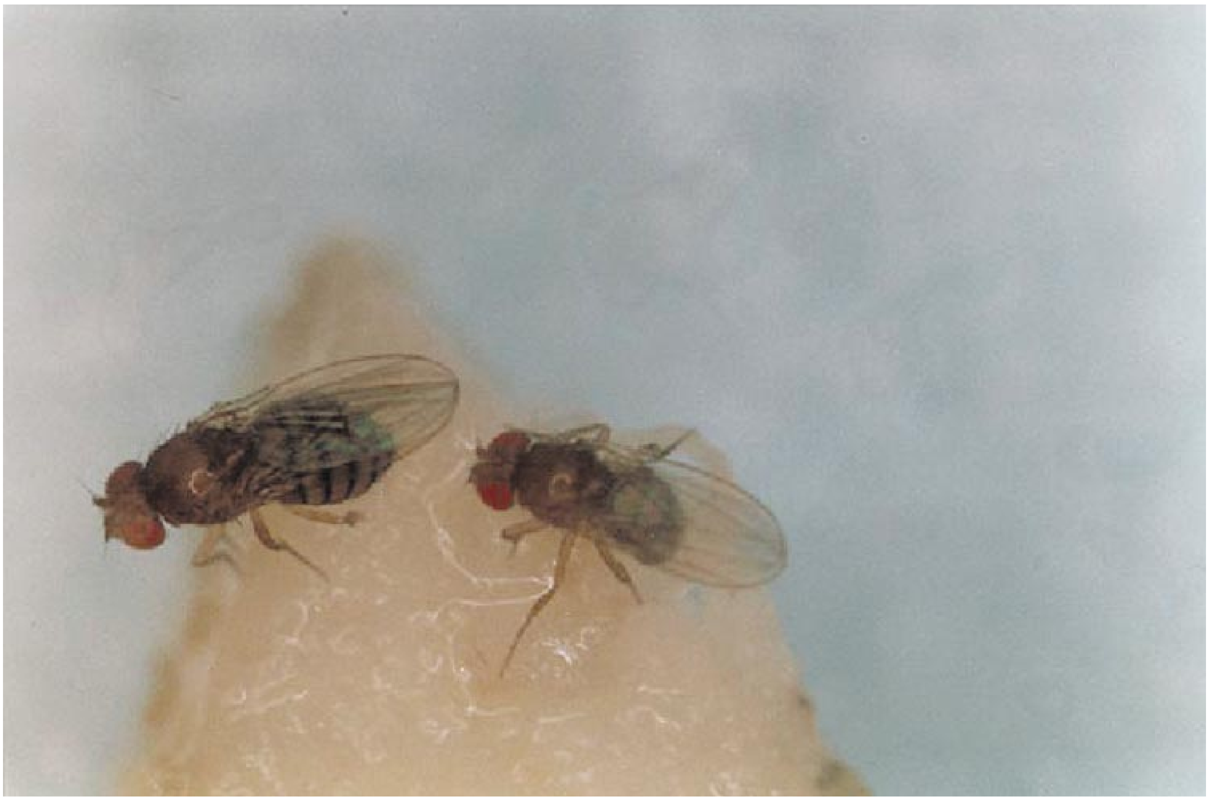

*Drosophila melanogaster* Reproduced courtesy of Tracey Chapman, The Galton Laboratory, University College London, UK

However, flies are not merely miniature humans, as they lack features such as an antibody response, cytoplasmic intermediate filaments and a myelin sheath.

### Source

The *Drosophila* Virtual Library (see Web resources).

### Tools for study

Any gene within the fruit fly genome can be mutated and subjected to detailed functional analysis within the context of an intact organism. The Berkeley *Drosophila* Genome Project (BDGP) has started a massive gene disruption project that uses individual, genetically engineered P transposable elements to target genes throughout the *Drosophila* genome (Spradling *et al.*, 1995); insertions from the collection now lie within or near most *Drosophila* genes (Spradling *et al.*, 1999). In addition, the inserted *P* elements in BDGP lines are mainly enhancer-traps (O'Kane and Gehring, 1987) that can often be used to acquire information about the expression pattern of disrupted genes or 'ep' insertions (Rørth, 1996) that can be used to direct ectopic expression of nearby genes that have not been structurally disrupted. These strains can be obtained from the *Drosophila* Stock Center, Bloomington, IN (see Web resources), and are invaluable for both forward and reverse genetics.

Expression patterns of genes in *Drosophila* embryos can be determined using the RNA *in situ* procedure developed by Tautz and Pfeifle (1989), which has been adapted to allow the screening of 96 RNA probes on whole-mount *Drosophila* embryos at the same time by Jasprien Noordermeer and Casey Kopczynski of Corey Goodman's laboratory at Berkeley. GFP-fusion genes can be used to look at gene expression in *Drosophila* embryos and egg chambers (Davis *et al.*, 1995).

The activity of enhancers can also be visualized in *Drosophila*. A P-element carrying the *S. cerevisiae* GAL4 transcription factor gene (P[GAL4]) is inserted randomly into the genome. The flies produced are crossed with flies expressing a reporter

Copyright © 2000 John Wiley & Sons, Ltd.

gene under the control of a promoter that is regulated by GAL4 (Brand and Perrimon, 1993). If the P-element has inserted downstream of an enhancer, the reporter gene expression seen in the fly will reflect the pattern of activity of the enhancer. Reporter genes can include not only the standard workhorse *lacZ*, but also genes that encode GFP which can be visualized in live flies, variants of GFP with different subcellular targeting signals, and proteins with any kind of desired biological activity that one wishes to express in a specific cell type.

Over 80 000 ESTs have been analysed at BDGP to find full-length cDNA clones for the *Drosophila* Gene Collection. Approximately 5800 non-redundant cDNA clones (representing ~40% of *Drosophila* genes) have been identified for single colony purification and re-arraying (Rubin *et al.*, 2000a). They aim to add more cDNA clones to a later version of this 'unigene' set. Version 1.0 should be available by 1 June 2000, as glycerol stocks in a 96- or 384- well format.

There are plans for a microarray service, a P-element mediated gene fusion project and a proteomics initiative at Cambridge University (see Web resources). A group at Stanford have generated microarray data for a time course of gene expression during the *Drosophila* life cycle (White *et al.*, 1999; see Web resources).

### Current status of genome knowledge

The ~180 Mb genome of *Drosophila* is stored as four chromosomes (X/Y, 2, 3 and 4). The majority of the 120 Mb of euchromatic, non-repetitive DNA is on chromosomes 2, 3 and X, with about 1 Mb on chromosome 4. The Y chromosome, in contrast, is almost entirely heterochromatin, as is most of the small chromosome 4, (Locke and McDermid, 1993). The genome has been completely sequenced (Adams *et al.*, 2000) and 13 601 genes have been predicted. ~38% of the predicted genes are supported by EST and protein matches, a further 39% are supported by just one of these types of evidence. The average predicted transcript size is ~3 kb with an average of four exons per gene. These are thought to be underestimates, due to the limited ability of the annotation programs to predict 3′ and 5′ untranslated regions, and the paucity of EST data for many such regions. There is significant variation in gene density, ranging from 0 to 30 genes per 50 kb, and, as in the human genome, gene-dense regions correlate with regions with higher GC content.

5536 of the genes are duplicated (which is a lower proportion of the proteome than is duplicated in the worm) and there are 8065 distinct gene families (Rubin *et al.*, 2000a). This 'core' proteome is surprisingly only twice the size of that of yeast and the same size as that of the worm. Whilst ~3000 genes were found to be common to fly, yeast and worm, 23% of fly proteins have no match from other organisms or Drosophila ESTs.

### FAQ—How can I get a *Drosophila* mutant of my favourite gene?

Publication of the fly sequence will tempt many people to consider obtaining and analysing a fly mutant of their favourite gene. A targeted homologous recombination system has only just been published (Rong and Golic, 2000). Given a friendly fly lab, this is well worth a try, although it remains to be seen how well it works generally. There are two main established alternatives: the first using RNA interference (RNAi) in precellular embryos to generate a mutant phenotype, and the second using P element gene disruption. Further options involving chemical mutagenesis may be considered by fly afficionados, and their power may increase in the future if molecular methods for detecting point mutations can be made more routine.

Injection of double-stranded RNA into precellular embryos leads to degradation of the corresponding endogenous RNA. For a guide, see **http://info.pitt.edu/~carthew/manual/Manual.html**. This is fast, good for embryonic phenotypes, but subject to the experimental variability involved in embryo injection, probably not as useful for postembryonic phenotypes, and doesn't yield a real mutant that can be used for rescue experiments and structure–function analyses. Attempts to use transgenic methods, such as expression of stem-loop RNA molecules, have so far had low success.

Alternatively, the P element collections can be used to find a real mutant. It still requires advice and help from a friendly fly lab, but for a fly lab it is (sometimes) fast and straightforward. It involves net-surfing, flypushing, and PCR.

- First, use your gene as a query in a BLAST search of the *Drosophila* sequence. To search

Copyright © 2000 John Wiley & Sons, Ltd.

against the whole genome, go to the NCBI Advanced BLAST search page: **http://www.ncbi.nlm.nih.gov/blast/blast.cgi?Jform = 1** and select '*Drosophila* genome' in the database box. This search should also tell you if the fly gene has been cloned. If you do identify a fly homologue of your gene, you should then BLAST this against non-*Drosophila* sequences. Your original gene should be the best, or ideally the only, high scoring hit in your pet organism. This will make it more likely that you identify the true *Drosophila* orthologue of your gene, rather than a more distantly related homologue.

- Next, you need to use your fly gene name, symbol or accession number in a search of the genes data at FlyBase (see Web resources) to see if there is an existing mutant of the fly orthologue that has already been characterized.
- If there is not, then the next step is to identify a mutant with a P insertion in or near to your fly orthologue.To do this, follow the FlyBLAST link on the BDGP homepage (see Web resources) and select 'P element insertion sites' from the 'dataset' pull down menu. You should use the fly genomic sequence of your gene, including a few kb (5 kb is usually sufficient) of upstream and downstream regions as your query sequence. P insertions in the transcribed region are likely to cause a mutation, those upstream of your gene can affect the expression of it. An alternative to BLASTing is to visualise P insertion sites and predicted genes using the GadFly link of FlyBase (see Web resources).
- P insertions near to your gene (within a few kb) can be 'remobilized'. This has the potential to induce imprecise excision of the insertion and flanking DNA, thus generating deletions. However, any such deletions would also include any genes between the transposon and your gene.
- However you identify your mutant, crossing heterozygous mutant flies will result in one-quarter of the progeny being homozygous for the mutation. These can be used to detect any phenotype. However, since there are only four chromosomes, these flies can be homozygous for as much as 40% of the genome, so flies that are trans-heterozygous for independently derived alleles should be used to eliminate most effects of unrelated recessive mutations on the same chromosome. It is also worth checking that the cloned gene can rescue any phenotype that is detected. Also, don't forget to verify your stock, e.g. by PCR, rather than just relying on the label!

### Future aims

Steve Russell (University of Cambridge, UK) is part of a team (with M. Ashburner, D. Glover and C. O'Kane) who have been awarded a grant of £3.5 million by the BBSRC Investigating Gene Function initiative, to establish core functional genomics resources for the UK *Drosophila* community. The resources they aim to provide can be divided into three categories; new mutant collections; microarray expression facilities; and proteomics facilities.

The group plan to generate a new collection of gene-trap P-element mutants using the GT1 vector devised in Daisuke Yamamoto's lab in Japan. Insertion 3′ to an active splice donor site will generate reporter gene fusion transcripts expressed in the same temporal and tissue-specific pattern as the host gene. In addition, they are collaborating with Gunter Reuter's lab in Halle to generate a second generation deletion kit utilizing the RS3 and RS5 elements developed by Kent Golic.

They will begin the FlyChip facility by generating slides containing the 'unigene' cDNA clones generated by the BDGP. They plan to compare the benefits of amplifying the entirety of each clone using a pair of vector specific primers, with using a vector-specific primer and a gene-specific primer to amplify the 3′ terminal 500 bp of each clone. The products will be arrayed on glass slides, in triplicate, using a commercial arraying robot. The slide will also include a set of non-*Drosophila* control clones to assess hybridization and reverse transcription efficiency. The group hope to be able to develop high quality normalization and control standards for the community. They are also planning to develop linear RNA amplification methods, for example the Eberwine system, to allow microarray screening from much smaller tissue samples. Ultimately, data will be deposited in the ArrayExpress database being developed at the EBI; however, they will develop an in-house database to allow local data mining. They are currently evaluating both commercially and publicly available analysis tools to find the most cost-effective solution. In the future, Russell envisages generating slides containing all of the predicted fly genes, and believes that the best way forward is via international collabora-

Copyright © 2000 John Wiley & Sons, Ltd.

tions to generate and store the requisite reagents. Similarly, he feels that the generation of whole genome tiles for analysis of DNA binding proteins and complexes will be best achieved by international cooperation to ensure uniformity and keep down costs. Ultimately, the goal of their microarray based approaches will be the analysis of gene expression at the single cell level. They envisage developments in both array generation and sample labelling over the next few years that will bring this goal within reach.

The Proteomics unit is being established jointly with the UK *Arabidopsis* community. The unit will separate proteins by 2D gel electrophoresis, using either conventional gels or the difference gel electrophoresis technique developed by Jon Minden at Carnegie Mellon in Pittsburg. Spots excised from the gels will be analysed by MALDI-TOF or tandem MS–MS mass spectrometry. Additionally, they will develop fractionation and immunoprecipitation techniques for protein complexes that will facilitate the purification, and analysis of macromolecular complexes from *Drosophila* tissues.

A steering group is being established that will regulate access to the facilities. UK fly workers will be invited to submit short proposals that will be evaluated by the steering group and prioritized. Preference will be given to researchers who make their data freely available quickly, bearing in mind that it is a condition of the award that all data be made freely available. At present the team favour a model whereby raw data is released onto their web server on a regular basis and individuals are able to download flat files of the images or raw data sets.

Rachel Drysdale, a senior curator of FlyBase, is one of a group of curators sited at Cambridge University Department of Genetics. During this year, FlyBase curators will be occupied with the reannotation that will be necessary to incorporate the data that BDGP will produce to complete the genome sequence. This will include rerunning BLAST searches, incorporating the new EST data being produced by BDGP and incorporating new data which is sent in daily to FlyBase, and other databases such as SWISSPROT, by individual researchers.

The fruit fly genome sequence has all been deposited in GenBank, EMBL and DDBJ and so it can be searched using the NCBI and EBI BLAST servers, or using the BLAST server provided by the BDGP. The Berkeley FlyBase group have developed two inter-related tools to view the data, 'GadFly' and 'GeneScene'. These are available directly from the FlyBase home page or from the BDGP home page. The GadFly database houses the gene annotations derived during the annotation of the genome sequence in the collaborative effort between Celera Genomics and the *Drosophila* Genome Projects. Each page includes a graphic of the gene showing intron/exon structure, a sketch of the flanking genes (each linked to their own Gadfly entry), a graphic of the InterPro protein domains in the gene and a section detailing the evidence upon which the annotation is based. There are links to GeneScene and to a form that readers can use to submit an update on the gene.

GeneScene is a genome browser that allows viewing of the gene in the context of its neighbouring genes in the genome. GeneScene can be launched from FlyBase, from GadFly or from the launcher at the BDGP site. GeneScene presents the sequence of one chromosome arm at a time as a horizontal scale bar. The genes are depicted above and below the bar, with their symbols and intron/exon structure. A colour coding system is used to indicate the type of evidence that supports the existence of each gene. Also shown are sequence gaps, repetitive elements, insertion sites of P elements from the BDGP gene disruption and mis-expression projects, and tRNAs.

Rachel Drysdale says that, as yet, FlyBase have no explicit plans for dealing with microarray data, since the technology to be used has not been fixed and there is not yet a great deal of data to work with. However, it is on the agenda for the next FlyBase project meeting, in October 2000, and there is talk of a common set of primers for the 13 000 genes, most likely to be organized by BDGP. The general feeling is that FlyBase will not be the database where the raw data is stored, but it will report the outcome of the analyses. She feels that the FlyBase team sited at Cambridge will be in an excellent position to collaborate with the microarray team of the UK functional genomics group as the technology develops over the next couple of years.

Meanwhile, FlyBase curators will be working to integrate the GadFly data with the FlyBase gene records and to relate the GadFly data with information about the molecular nature of the genes that exists in the literature. They also plan to continue their curation of literature covering

Copyright © 2000 John Wiley & Sons, Ltd. 

genetic and phenotypic studies and to keep the site under continual review to keep abreast of how best to serve their users.

### Web-based resources

### General information on *Drosophila*

**The Interactive Fly**
**http://flybase.bio.indiana.edu/allied-data/lk/interactive-fly/aimain/1aahome.htm**
This site has lists of *Drosophila* genes by name, by functional category and by biochemical pathway. There are also pages with information on developmental stages and which genes are active in each stage or involved in the formation of particular organs. Authors: Tom Brody and Judith Brody.

**The *Drosophila* Virtual Library**
**http://www.ceolas.org/fly/index.html**
This site is part of the 'Model Organisms' group within the 'Biosciences' area of the World-Wide Web Virtual Library. The 'What is *Drosophila*?' link takes you to an excellent introduction to *Drosophila*, which has formed the basis of the 'Background' section of this article. There is also an extensive collection of links to *Drosophila* web sites and a list of books on *Drosophila*, which is included below. Author: Gerard Manning.

### Databases and sequencing projects

**FlyBase**
**http://flybase.bio.indiana.edu/**
This site has mirrors in the UK, France, Australia, Japan, Israel and Taiwan. The site (The FlyBase Consortium, 1999) provides many services, including cytological maps and searches of genes or alleles. Users can browse functional, structural and expression data for *Drosophila* genes. Searches of polypeptides and transcripts are also available. There are links to the two genome projects (BDGP and EDGP), an EST search and a listing of EST-gene links. Users can search for stocks and order them, and browse the homepages of two stock centres. There are also searchable databanks of transposon vectors, transposon insertions and aberrations and a collection of body part images and terms. Searches of literature references and an address list of *Drosophila* researchers are also available on the site. There is a Bionet newsgroup dedicated to *Drosophila* (bionet.*Drosophila*). Users can search the archives of messages from this site.

**Berkeley *Drosophila* Genome Project (BDGP)**
**http://www.fruitfly.org/**
Offers BLAST and text searches of the fly DNA and protein sequence data, map viewers of chromosomes or chromosome arms, and other analysis tools such as gene and regulatory sequence prediction programs. The ESTs page allows searches for a particular EST, for the results of BLAST searches with an EST, and for the expression patterns of ESTs in *Drosophila* embryos. There is also a collection of protocols in use at BDGP.

**European *Drosophila* Genome Project (EDGP)**
**http://edgp-dev.ebi.ac.uk/**
The EDGP is a consortium of that are sequencing the X chromosome of *Drosophila melanogaster*. The site contains information on the clones sequenced. The sequence data is available in raw and annotated formats and can be obtained by FTP. BLAST searches against the EDGP and BDGP genome data, transposons, repeats, ESTs or proteins are available to users. Author: Panayiotis (Takis) Benos.

***Drosophila* Chromosome 4 Site**
**http://www.biology.ualberta.ca/dros4.hp/4th_main.htm**
This page links to Dr John Locke's homepage: **http://gause.biology.ualberta.ca/locke.hp/locke.html** which has a lot of background on chromosome 4 but, as yet, no sequence data. Author: John Locke.

### *Drosophila* stock centres

**The Bloomington *Drosophila* Stock Center**
**http://flystocks.bio.indiana.edu/**
This site has information to help users submit and order stocks, there are options to search or browse the list of stocks held. There are also links to FlyBase, to a selection of other *Drosophila* web resources and to the homepages of other stock centres. There is even advice on getting rid of flies, for those occasions when they escape!

**The Szeged *Drosophila melanogaster* P Insertion Mutant Stock Centre**
**http://gen.bio.u-szeged.hu/stock/**
The centre has recessive lethal P insertion mutants on chromosome 2 and 3 and EP insertion mutants for mis-expression lines.

Copyright © 2000 John Wiley & Sons, Ltd. 

### Functional genomics projects

**_Drosophila_ DNA Microarray Homepage**

**http://cmgm.stanford.edu/~kpwhite/index.html**

This page contains protocols for making *Drosophila* arrays. Author: Kevin White.

**http://quantgen.stanford.edu/**

This page gives access to the data generated so far (White *et al.*, 1999), a time course of gene expression during the *Drosophila* life cycle.

**Functional Genomics for UK Drosophilists**

**http://www.gen.cam.ac.uk/~flychip/**

This page contains details of the planned functional genomics projects to be run at Cambridge University. These are microarray analyses of gene expression, a genome-wide P element-mediated GAL4 gene fusion project and a proteomics initiative. Author: Steve Russell.

**FlyTrap**

**http://brainbox.gla.ac.uk/flytrap/**

An interactive database for displaying gene expression patterns, in particular P[GAL4] patterns, via an intuitive WWW based interface. In particular the site concentrates on patterns of expression in the *Drosophila* brain. Basic searching is now available and, since the search page uses JavaScript, it will work off-line from a CD-ROM copy. Authors: J. Douglas Armstrong, Chris Edwards and Kim Kaiser.

**FlyView**

**http://pbio07.uni-muenster.de/**

FlyView is an image database, which is mainly composed of expression patterns of genes from enhancer trap lines and cloned genes. The database is designed to be compatible with FlyBase. The main aim is for users to be able to compare images on their computer screens and to search for special patterns at different developmental stages, so all of the images are accompanied by text descriptions that can be used for. Author: Wilfried Janning.

**The Gene Interactions in the Fly Transworld Server (GIFTS)**

**http://gifts.univ-mrs.fr/index.html**

This site gives access to three databases: FlyNets (Sanchez *et al.*, 1999), a database of molecular interactions (protein–DNA, protein–RNA, protein–protein) in *Drosophila*; SOS–DGDB (Sites on Sequences–*Drosophila* Genes DataBase), a collection of *Drosophila* protein binding sites which have been annotated on the DNA sequence; and KNIFE, a prototype of a database of *Drosophila* developmental regulatory networks. There is also an impressive set of links to BLAST servers on the web. Author: Bernard Jacq.

### *Drosophila* books

### General

*The Making of a Fly* (Peter Lawrence; Blackwell Scientific, Oxford, 1992).

*Drosophila* (Brian Shorrocks; Ginn & Co, London, 1972)

*The Development of Drosophila melanogaster* (ed. Michael Bate & Alfonso Martinez-Arias; Cold Spring Harbor Laboratory Press, New York, 1993)

*Biology of Drosophila* (ed. M. Demerec; Cold Spring Harbor Laboratory Press, New York, 1994).

*The Embryonic Development of Drosophila* melanogaster (JA Campos-Ortega & V Hartenstein; Springer-Verlag, Berlin/Heidelberg, 1985)

### Techniques

*Drosophila, A Practical Approach* (ed. DB Roberts, IRL Press, Oxford, 1998).

*Drosophila, A Laboratory Manual* (Michael Ashburner, Cold Spring Harbor Laboratory Press, New York, 1989)

*Drosophila melanogaster: Practical Uses in Cell and Molecular Biology* (ed. Lawrence SB Goldstein & Eric A Fryberg; Academic Press, New York, 1994)

*Fly Pushing: The Theory and Practice of Drosophila Genetics* (Ralph J Greenspan, Cold Spring Harbor Laboratory Press, New York, 1997)

### Source

The *Drosophila* Virtual Library (see Web resources).

### References

Adams MD, Celniker SE, Holt RA, Evans CA, Gocayne JD, Amanatides PG. 2000. The genome sequence of *Drosophila melanogaster*. *Science* **287**: 2185–2195.

Bainton RJ, Tsai L T-Y, Singh CM, Moore MS, Neckameyer WS, Heberlein U. 2000. Dopamine modulates acute responses

Copyright © 2000 John Wiley & Sons, Ltd. 

to cocaine, nicotine and ethanol in *Drosophila*. *Curr Biol* **10**: 187–194.

Brand A, Perrimon N. 1993. Targeted gene expression as a means of altering cell fates and generating dominant phenotypes. *Development* **118**: 401–415.

Davis I, Girdham CH, O'Farrell PH. 1995. A nuclear GFP that marks nuclei in living *Drosophila* embryos; maternal supply overcomes a delay in the appearance of zygotic fluorescence. *Dev Biol* **170**(2): 726–729.

Fortini ME, Bonini NM. 2000. Modeling human neurodegenerative diseases in *Drosophila*: on a wing and a prayer. *Trends Genet* **14**: 161–167.

Hendricks JC, Finn SM, Panckeri KA, Chavkin J, Williams JA, Sehgal A, Pack AI. 2000. Rest in *Drosophila* is a sleep-like state. *Neuron* **25**(1): 129–138.

Kandel E, Abel T. 1995. Neuropeptides, adenylyl cyclase, and memory storage. *Science* **268**: 825–826.

Krumlauf R. 1992. Evolution of the vertebrate Hox homeobox genes. *Bioessays* **14**: 245–252.

Li H, Chaney S, Forte M, Hirsh J. 2000. Ectopic G-protein expression in dopamine and serotonin neurons blocks cocaine sensitization in Drosophila melanogaster. *Curr Biol* **10**: 211–214.

Locke J, McDermid HE. 1993. Analysis of *Drosophila* chromosome 4 using pulsed field gel electrophoresis. *Chromosoma* **102**: 718–723.

O'Kane CJ, Gehring WR. 1987. Detection *in situ* of genomic regulatory elements in *Drosophila*. *Proc Natl Acad Sci USA* **84**: 9123–9127.

Pawson T, Bernstein A. 1990. Receptor tyrosine kinases—genetic evidence for their role in *Drosophila* and mouse development. *Trends Genet* **6**: 350–356.

Potter CJ, Turenchalk GS, Xu I. 2000. *Drosophila* in cancer research—an expanding role. *Trends Genet* **16**(1): 33–39.

Rong YS, Golic KG. 2000. Gene targeting by homologous recombination in *Drosophila*. *Science* **288**: 2013–2018.

Rørth P. 1996. A modular mis-expression screen in *Drosophila* detecting tissue-specific phenotypes. *Proc Natl Acad Sci USA* **93**: 12418–12422.

Rubin GM, Hong L, Brokstein P, Evans-Holm M, Frise E, Stapleton M, Harvey DA. 2000a. A *Drosophila* complementary DNA resource. *Science* **287**: 2222–2224.

Rubin GM, Yandell MD, Wortman JR, Gabor Miklos GL, Nelson CR, Hariharan IK, *et al.* 2000b. Comparative genomics of the eukaryotes. *Science* **287**: 2204–2215.

Sanchez C, Lachaize C, Janody F, Bellon B, Röder L, Euzenat J, Rechenmann F, Jacq B. 1999. Grasping at molecular interactions and genetic networks in *Drosophila* melanogaster using FlyNets, an Internet database. *Nucleic Acids Res* **27**(1): 89–94.

Shaw PJ, Cirelli C, Greenspan RJ, Tononi G. 2000. Correlates of sleep and waking in *Drosophila melanogaster*. *Science* **287**: 1834–1837.

Spradling AC, Stern D, Beaton A, Rhem EJ, Laverty T, Mozden N, Misra S, Rubin GM. 1999. The Berkeley *Drosophila* Genome Project gene disruption project: single P-element insertions mutating 25% of vital *Drosophila* genes. *Genetics* **153**(1): 135–177.

Spradling AC, Stern D, Kiss I, Roote J, Laverty T, Rubin GM. 1995. Gene disruptions using P transposable elements: an integral component of the *Drosophila* genome project. *Proc Natl Acad Sci U S A* **92**: 10824–10830.

Tautz D, Pfeifle C. 1989. A non-radioactive *in situ* hybridization method for the localization of specific RNAs in *Drosophila* embryos reveals translational control of the segmentation gene hunchback. *Chromosoma* **98**: 81–85.

The FlyBase Consortium. 1999. The FlyBase Database of the Drosophila genome projects and community literature. *Nucleic Acid Res* **27**: 85–88.

White KP, Rifkin SA, Hurban P, Hogness DS. 1999. Microarray analysis of *Drosophila* development during metamorphosis. *Science* **286**: 2179–2184.

---

*Comparative and Functional Genomics* is a cross-organism journal, publishing studies on complex and model organisms. The 'Featured Organism' article aims to present an overview of an organism, primarily for those working on other systems. It provides background information on the organism itself and on genomics studies currently in progress. It also gives a list of websites containing further information and a summary of the status of the study of the genome. These sections are a personal critical analysis of the current studies of the particular organism. The 'Future Aims' section is intended to be of interest to readers who work on the chosen organism and those who study other systems, and the opinions expressed therein are those of the named contributors.

Many thanks to Dr Steve Russell for sharing his thoughts on the future of *Drosophila* genomics research and to Dr Rachel Drysdale for sharing her thoughts on the future of FlyBase.

**Dr Steven Russell**
Department of Genetics, University of Cambridge, Downing Street, Cambridge, CB2 3EH, UK.
Fly functional genomics: **http://www.fly.to/genomics** FlySox: **http://www.gen.cam.ac.uk/~sr120**

**Rachel Drysdale**
FlyBase (Cambridge), Department of Genetics, University of Cambridge, Downing Street, Cambridge CB2 3EH, UK

Copyright © 2000 John Wiley & Sons, Ltd. 
